# Regenerated Fiber’s Ideal Target: Comparable to Natural Fiber

**DOI:** 10.3390/ma17081834

**Published:** 2024-04-16

**Authors:** Guohongfang Tan, Tianshuo Jia, Zhenzhen Qi, Shenzhou Lu

**Affiliations:** National Engineering Laboratory for Modern Silk, College of Textile and Clothing Engineering, Soochow University, Suzhou 215123, China; 20234015007@stu.suda.edu.cn (G.T.); 20215215010@stu.suda.edu.cn (T.J.); 20224015005@stu.suda.edu.cn (Z.Q.)

**Keywords:** silk fibers, protein, biomimetic, regenerated spinning, liquid crystal

## Abstract

The toughness of silk naturally obtained from spiders and silkworms exceeds that of all other natural and man-made fibers. These insects transform aqueous protein feedstocks into mechanically specialized materials, which represents an engineering phenomenon that has developed over millions of years of natural evolution. Silkworms have become a new research hotspot due to the difficulties in collecting spider silk and other challenges. According to continuous research on the natural spinning process of the silkworm, it is possible to divide the main aspects of bionic spinning into two main segments: the solvent and behavior. This work focuses on the various methods currently used for the spinning of artificial silk fibers to replicate natural silk fibers, providing new insights based on changes in the fiber properties and production processes over time.

## 1. Introduction

Spiders are widespread in nature as specialized species capable of naturally secreting protein fibers [1,2]. The mechanical strength of spider silk is unique in nature and it is one of the toughest materials known to date [3,4]. Spider silk has similar strength to steel and significantly higher strength than silk, rubber, and other synthetic fibers. Its elongation at breaking point is almost the same as that of silk and other synthetic fibers and much higher than that of steel and Kevlar fibers; its breaking energy is also several times that of Kevlar [5] (Table 1). However, spiders cannot be farmed on a large scale, and the production of natural spider silk fibers is very limited, making it difficult to produce them in large quantities for use as conventional materials [4,6]. Therefore, researchers in various countries have sought to develop artificial spider silk that is similar to the natural fiber.

Genetic engineering technology has played a great role in the artificial synthesis of spider silk proteins, and research on their synthesis using transgenic technology has developed rapidly [1,7,8,9,10]. Huemmerich et al. [11] developed a baculovirus expression system that can be applied to the expression of silk genes in insect cells. The proteins found in the spider glands were translationally modified to increase the solubility of the resulting proteins, allowing secretion-encoding signals for wasp proteins, demonstrating that insect cells are a promising resource for silk protein production. Fahnestock et al. [12] tested the expression of yeast Pichia pastoris as a synthetic silk gene and noted that it was expressed in a similar manner to that in bacteria, with reasonable protein yields. However, the experimental follow-up evidenced some difficulties in completing the protein purification process due to excessive aggregation. Zhang et al. [7] used a CRISPS/Cas9-initiated targeting strategy to integrate the spider silk protein gene into the Bombyx mori genome, obtaining exogenous protein expression. This study demonstrates the possibility of using the silkworm as a spider gene vector for the industrial production of high-performance fibers. As of 2023, the majority of the research has focused on natural spider silk, while relatively little research has been conducted on regenerated spider silk. This could be due to the difficulties in collecting natural spider silk fibers, limited production capabilities, etc. Thus, at present, the data obtained remain unsatisfactory. This has led to a significant question among scientists: how can we obtain strong, fine fibers on a larger scale?

**Table 1:** Mechanical data for silk and other materials.

Fiber Type	Strength (MPa)	Toughness (MJ/m^3^)	Strain (%)	Refs.
*B. mori* cocoon silk ^1^	300–740	70	70	[13,14]
*B. mori* reeled silk ^2^	700	150	18	[15]
*L. hespeus* ^3^	1400 ± 100	243 ± 29	30	[16,17]
*A. diadematus* ^4^	1700 ± 200	225 ± 29	165 ± 15	
*N. edulis* ^5^	1200 ± 200	215 ± 36	/	[18,19]
Nylon	950	80	18	[20]
Kevlar	3600	50	2.7	
Carbon fiber	4000	25	1.3	
Polypropylene	490	/	23	[21]

^1, 2^ *B. mori* cocoon silk and *B. mori* reeled silk are obtained from silkworm. ^3^ *L. hespeus* is obtained from black widow spider. ^4^ *A. diademats* is obtained from garden spider. ^5^ *N. edulis* is obtained from tropical spider.

Silkworms, as metamorphic insects, form cocoons when they reach the fifth instar and spit out silk through their serous glands to wrap themselves in a pupa. The main component of the pupa is silk fiber, which is a valuable resource [22]. Silk is obtained at room temperature, under normal pressure, using aqueous solutions of proteins [23]. Although the comprehensive performance of silk is inferior to that of spider silk, it still demonstrates good performance that is superior to that of other natural fibers. Unlike spiders, silkworms are peaceful, allowing for large-scale production [24]. In China, silk is used for international exchange as a valuable clothing material, and the history of the use of silk as a raw textile material spans more than 5000 years. Among all silk types produced by silkworms, domestic silk, quassia silk, and castor silk are the main silks used as textile materials. Domestic silk is used as the main raw material for silk production. Due to its 99% protein content, the production process is sustainable and eco-friendly and it can be easily recycled, indicating silk’s potential for use in composites in engineering and medicine. More importantly, silk and its biologically highly evolved production methods bring new inspiration and templates for the study and preparation of new high-performance fiber silks in the modern world, where increasing attention is being paid to renewable energy sources and energy efficiency [25,26,27,28].

At present, the industry faces a dilemma whereby the silk from silkworms is high in quantity and poor in quality, while spider silk is low in quantity and high in quality [29]. The amino acid compositions of silk from different spider species and *B. mori* sericin are summarized in Table 2. We can see that the different types of spider and silkworm silk exhibit some differences in their amino acid compositions, but, in general, the main amino acid components are glycine, alanine, glutamic acid, and serine. The amino acid content of small side groups in spider silk is generally much lower than that of silk sericin, so the degree of regularity of the molecular arrangement in spider silk is also smaller than that in silkworm silk. However, since the content of polar amino acids in spider silk is much larger than that in silkworm silk, there is a large molecular force between the molecular chains, even if they exhibit a non-regular arrangement. This may be one of the reasons why the mechanical properties of spider silk are superior to those of silkworm silk. Researchers have attempted to improve the yield and quality of recombinant and regenerated silk proteins via synthetic means. Regenerated silk protein has become a major focus of research; it is particularly important to ensure that the structure of the silk protein’s molecular chain is not destroyed and that it is uniformly distributed in the solution for subsequent spinning. Moreover, it is desirable to obtain a series of controllable high-performance silk-based fibers that can be directly used in specific application scenarios, such as surgical sutures, wound dressings, tissue engineering scaffolds, and so on, which introduces new requirements for the spinning process.

We provide a brief description of the composition of silk protein and the silkworm’s natural spinning process, discuss the most common methods of dissolving silk fibroin (SF) at present, describe the various methods used to spin artificial silk fibers at present, and provide insights into the fiber properties and the trends in the production process over time.

## 2. SF Composition

### 2.1. Primary Structure

The amino acid chain of SF is similar to that of other protein substances with a linear structure whose molecular weight ranges from 20 k to 400 k [36,37]. The majority of the amino acid sequence is composed of glycine (Gly), alanine (Ala), and serine (Ser), which constitute the crystalline region of SF, representing approximately 85% of its total composition [38,39]. Amino acids with large side groups, such as phenylalanine (Phe), tyrosine (Tyr), and tryptophan (Try), constituting the non-crystalline region, together form the primary structure of SF [24,40].

**Figure 1:**
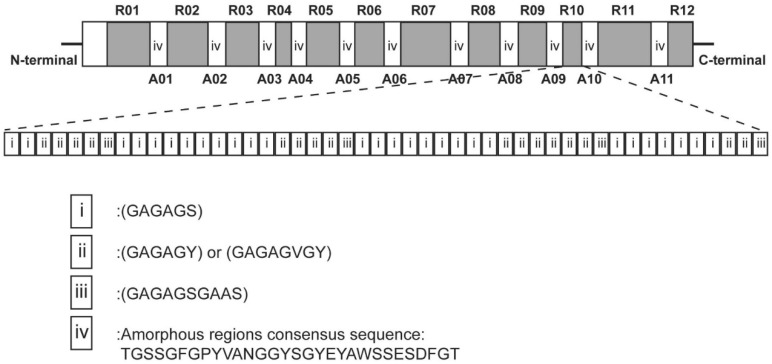
A schematic representation of the fine organization of the primary structure of the *B. mori* SF heavy chain. R01···R12 and A01···A11 represent the unique arrangements of 12 repetitive and 11 amorphous regions, respectively. The approximate amino acid sequence of the R10 region is illustrated, containing i, ii, and iii. Module iv represents a typical amino acid sequence in the amorphous region. Copyright 2005 American Chemical Society, reproduced with permission from [49].

Using cDNA technology, it has been found that SF is composed of three main types of subunits: the heavy chain (H chain), the light chain (L chain), and glycoprotein P25 (P25) [41,42,43]. The H chain exists as the SF’s main chain [44,45], with an average molecular weight of approximately 390 kDa [46]. It consists of 45.9% glycine, 30.3% alanine, 12.1% serine, 5.3% tyrosine, 1.8% valine, and 4.7% of the other 13 amino acids [47]. Organizationally, the heavy chain is a highly regular biopolymer consisting of 12 hydrophobic and 11 hydrophilic structural domains [48]. Its amino acid sequence can be roughly divided into four modules: i, ii, iii, and iv [49], which is described in Figure 1. Module i consists of highly repetitive GAGAGS sequences [50,51], forming the crystalline region of the silk fiber; the total number of repeats of this sequence within the SF is 433 [52]. Because the total number of amino acid residues in the heavy chain is 5263, it follows that almost half of the amino acid sequence within the heavy chain consists of the repetitive AGSGAG sequence. Module ii mainly contains three types of sequences: GAGAGY, GAGAGV, and GAGAGVGY [53]. Module ii has fewer repeats in the amino acid chain and mostly contains hydrophobic and/or aromatic residues. The sequences of module iii and module i are similar, with the only difference being the presence of the AAS sequence in module iii [54]. Module iv contains sequences with negatively charged, polar, broadly hydrophobic, and/or aromatic residues, such as TGSSGFGPYVANGGYSGYEYAWSSESDFGT. Such disordered structures act as links connecting the individual crystallographic regions and constitute amorphous regions inside the heavy chain. Regarding the H chain, some scholars have proposed another view: the molecular weight of the H chain from SF is considered to be smaller, at 350 kDa [55]. Non-repetitive ends may play a role in the spinning process and in initiating protein assembly [56,57,58]. This claim needs to be further verified.

The L chain has an average molecular weight of 28 kDa [29]. Its Cys residue at position 190 is bound to the Cys residue at the inverse position 20 of the H chain in a disulfide bond [59]. P25 has an average molecular weight of 25 kDa and is linked to the H and L chains via hydrophobic interactions [60,61]. Zhou et al. [62] combined the shotgun procedure, currently applied in genome sequencing, with a traditional physical map-directed sequencing strategy; based on the sequences of the SF genes and encoded proteins, they obtained the complete amino acid sequence of SF.

### 2.2. Secondary Structure

**Figure 2:**
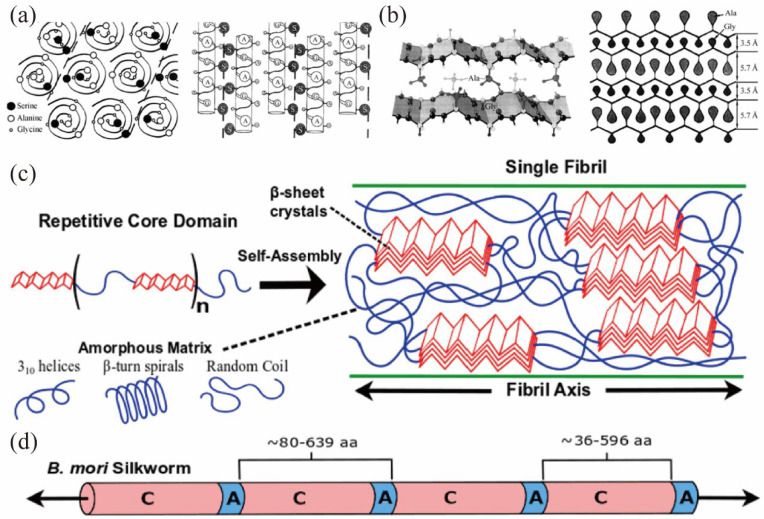
A schematic representation of the secondary sequence of SF. (**a**) α-helix structure [59]. (**b**) β-sheet structure [55]. (**c**) A schematic of SF’s primary sequence, showing a repetitive core domain consisting of alternating rigid and flexible blocks. The self-assembly of SF results in the formation of nanocrystals, which consist of “stacked” β-sheets with hydrophobic interactions between amino acid side chains extending orthogonally from the sheet plane, embedded in a hydrophilic amorphous matrix [56]. (**d**) The *B. mori* SF consists of A-C repeats ranging from 80 to 639 amino acids, where C is rich in glycine-X motifs [56].

The secondary structure of a protein refers to the specific conformation of the amino acids according to the backbone atoms of the main chain, which are coiled or folded along a certain axis [63]. The secondary structure contains the conformational units of the complete peptide chain conformation (space structure) and is the basis for the complex spatial conformation of proteins [63]. The determination of the orientation and structure of the polypeptide chain is required to obtain a complete view of the organization of the SF molecule [63]. The data on the spatial structure of SF, however, remained relatively limited until the 21st century. Figure 2 describes the SF’s secondary sequence with schematic representation.

M. Shimizu [64] first discovered two secondary structures in SF, the α-type and β-type, which are considered to be the two main structures constituting the secondary structure of solid SF. Kratky [65] experimentally confirmed the existence of both of these crystalline forms and suggested that the α-type in SF was different from the α-helical structure found in proteins in general. As a result of the efforts of several scholars, the secondary organization of SF can be considered to exist in four states [66]: random [67], α-helix [68,69], β-turn [70], and β-sheet [71].

The random structure is distinguished from the other three secondary structures as an amorphous region in SF [72]. The molecular chains are uncrosslinked in this region, ensuring room for other crystalline regions to move while the fiber is being stretched or contracted. This may be one of the fundamental reasons that silk fibers themselves have superior toughness and strength. The α-helix structure consists of an alanine-rich amino acid sequence. The vast majority consists of seven types of amino acid sequences as the base sequences, where positions A and D tend to be occupied by hydrophobic residues, while other positions are occupied by hydrophilic residues, and this seven-axis module tends to form a coiled α-helix structure [73]. The β-sheet structure consists of fully extended polypeptide chain segments, interacting with each other through the strength of the hydrogen bonds between -NH and C=O in neighboring chain segments [74]. The β-turn structure can be achieved by arranging the 36 amino acids with β-turning conformations in parallel and assigning intermolecular hydrogen bonds between the carbonyl oxygen of an even number of residues in each chain and the amino proton of the closest glycine in the neighboring chain [75].

### 2.3. Methods to Resolve SF’s Secondary Structure

There are many ways to resolve the structure of SF, such as nuclear magnetic resonance imaging (NMR) [66,76,77,78], X-ray diffraction (XRD) [79,80,81,82], electron diffraction [80,83,84], laser Raman spectrometry (Ram) [2,85,86], and Fourier transform infrared spectroscopy (FTIR) [87,88,89].

Compared to other secondary structure determination methods, amino acid side chain uptake provides valuable information in studying the mechanisms of protein reactions, due to the fact that the side chains are often central to the molecular reaction mechanisms [90,91,92]. FITR represents the most efficient method to resolve the secondary structure of SF [93,94]. With FITR, environmental and structural changes can be inferred in order to understand the molecular reaction mechanism [95]. In order to facilitate the exploration of the structure of SF by subsequent researchers, the distribution of the unique protein peptide chains of SF has been investigated. The general categorization is based on the range of intervals presented in the spectra: amide I (1700–1600 cm^−1^), amide II (1600–1500 cm^−1^), amide III (1300–1200 cm^−1^), and amide IV (<1200 cm^−1^) [96]. The content of each secondary structure can be accurately calculated via peak fitting [97]. Due to the different experimental environments and equipment used, the vibration spectral bands are also shifted to some extent, so that they are attributed to certain intervals [97,98]. Table 3 illustrates the interval distribution of the secondary structures of SF under FITR. Amide I is delineated as follows: 1647–1655 cm^−1^ for random, 1656–1662 cm^−1^ for the α-helix; 1622–1627 cm^−1^ for the β-sheet [44]. Amide Ⅱ is delineated as follows: 1534–1548 cm^−1^ for random; 1529–1533 cm^−1^ for the β-sheet [98,99]. Amide III is delineated as follows: 1240–1230 cm^−1^ for random; 1263–1265 cm^−1^ for the β-sheet [100].

Xiao Hu et al. [100] first detected the glass transition temperature of SF during heating by using differential scanning calorimetry (DSC), thermoregulation (TMDSC), and FTIR time-resolved techniques, demonstrating that the water bound between molecules acts as a plasticizer and strongly affects the secondary structure of SF. The DSC study revealed that, during rapid heating, SF displayed a water-induced glass transition at around 80 °C, which was caused by the temporary binding of the water–SF structure. Moreover, the temperature-dependent FTIR and TMDSC techniques are important for the study of water’s effects on polymers.

In addition to FTIR, we can also use the Raman method to determine the type and distribution of SF’s secondary structures [101]. Unlike the FTIR method, the Raman method is based on the Raman scattering effect [102]. When light propagates through SF, it interacts with the SF and changes the frequency of the light. The scale of the data at specific detection wavelengths in Raman reflects the microenvironment of the amino acid domains [103]. P. Monti et al. [104] reported the complete Raman data spanning 1800–200 cm^−1^ for silk Ⅰ-Cp for the first time. Shao et al. [96] used the Raman method to determine the protein conformation and orientation of the mulberry silkworm species *B. mori* and *S. Cynthia* ricini and the spider species *N. clavipes* and *N. edulis*, on the basis of data on the amide I bands. Compared to FTIR, the useful detection range of the Raman method covers mainly amide I and amide III. Table 4 illustrates the interval distribution of SF’s secondary structures under Raman. Amide I is delineated as follows: 1660–1665 cm^−1^ for random; 1645–1658 cm^−1^ for the α-helix; and 1665–1680 cm^−1^ for the β-sheet [105]. Amide Ⅲ is delineated as follows: 1250 cm^−1^ for random; 1264–1310 cm^−1^ for the α-helix; and 1220–1245 cm^−1^ for the β-sheet [85].

### 2.4. Spatial Structure

**Figure 3:**
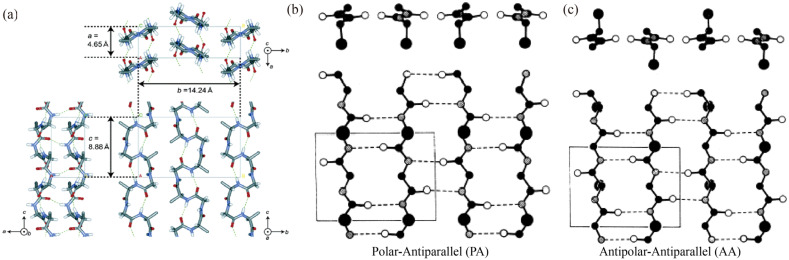
Model of SF’s spatial structure. (**a**) Stacked structures obtained from atomic least squares (LALS) calculations for silk Ⅰ. The dashed lines indicate hydrogen bonds [117]. (**b**) Parallel β-sheet lamellar structure [56]. (**c**) Antiparallel β-sheet lamellar structure [106]. Copyright 1997 John Wiley and Sons, reproduced with permission from [117].

The spatial structure of SF was first mentioned in a 1955 study [56]. By means of XRD, two crystal types can initially be observed, silk I and silk II [106,107]. This also serves as a general method of categorizing the hydrophilic and hydrophobic structure of SF. Figure 3 describes the SF’s Silk I and silk II structure. Silk III’s structure was discovered later; it exists in the regenerated silk film formed at the air–water interface and belongs to the tripartite crystal system resulting from silk protein crystallization [108]. Silk Ⅰ was first found within air-dried silk threads, generally considered to be abundant in liquid silk proteins stored by silkworms prior to spinning. It exists in a sub-stable crystalline state between the α-helix and β-sheet. Lotz et al. [109] proposed a “crankshaft” molecular model based on peptide diffraction. Later, Konishi et al. [110] proposed a loose four-helix model, and Fossey et al. [71] proposed a lamellar structure model, but cannot comprehensively explain the silk Ⅰ structure. It is now more commonly considered to be a type I repeating β-turn structure [60,111]. Compared to silk I, silk II is more mature and well studied. The silk II structure consists of a regular antiparallel β-sheet structure composed of polypeptide chain segments with very little or no side groups of amino acids. These peptide chain segments are regularly ordered and hydrogen-bonded; they exist in the lowest state energetically and remain stable. Silk II and silk Ⅰ can be interconverted under certain conditions, providing SF with good tunability in terms of its physical properties. Valluzzi observed that the crystal structure of silk III is similar to that of the corresponding glycine II, which is less abundant and unstable, being a uniaxially oriented crystalline structure with a triangular cross-section and a left-handed 2/3 helix reassembled into a hexagonal shape [60,112].

The tiny crystalline zones of silk proteins are uniformly distributed in continuous amorphous intervals; thus, they have good elasticity and a high tensile modulus, while maintaining a certain degree of strength in the fiber. However, SF’s membranes lose this property due to drying and organic solvents, probably because the crystalline–amorphous structure of SF is disrupted during secondary processing.

SF itself can be considered a supramolecular polymer. Its properties are affected by non-covalent intermolecular interactions, which can also explain the super-strong mechanical properties of the fibers, which are formed through non-covalent interactions such as hydrogen bonding and hydrophobic interactions [113,114]. This supramolecular structure is the basis for the ultra-high strength and robustness of silk, while the non-covalent interactions of the fibers themselves provide them with a certain self-repairing ability. This can be explained by the shrinkage phenomenon [5,115,116].

## 3. Eco-Spinning

The natural silk spitting process of the silkworm is a gentle and efficient process. It can be performed at lower spinning speeds and ambient temperature and pressure in aqueous solvent system environments. Current research suggests that this may be due to the liquid crystal spinning method practiced by the silkworm, rather than the isotropic solution spinning method. In this process, under the synergistic effect of stress and different types of metal elements, the molecular chain of SF is gradually changed from an α-helix or random conformation to a β-sheet conformation, realizing the transformation from liquid SF to solid silk.

**Figure 4:**
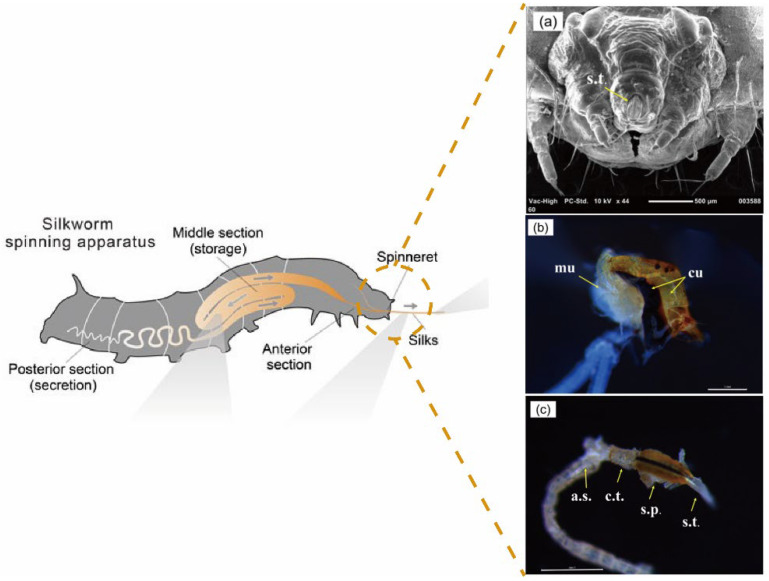
Silkworm spitting device [63]. (**a**) The spinneret with cuticles and muscles [118]. (**b**) The spinneret without cuticles and muscles, mu = muscles, cu = cuticles [118]. (**c**) c.t. = common tube, s.p. = silk press, s.t. = spinning tube, a.s. = anterior silk gland. Scale bar = 1 mm [118].

SF is first synthesized in the epithelial walls of the serous glands, at which point the concentration of the SF solution obtained is approximately 12 wt%. Subsequently, as the external muscles of the serous glands are squeezed, the solution moves forward in the vesicle/pot belly, while the surfaces of the glands absorb water, resulting in a gradual increase in the solute concentration to 25 wt%. When it enters the middle silk gland, it mixes with the sericin secreted by the epithelial wall and forms a preliminary package, at which point the left and right SF are evenly wrapped by the sericin and move toward the anterior silk gland as the muscle squeezes and stretches. As the diameter of the anterior silk gland decreases, the fiber’s fineness gradually decreases. Finally, the silk is held in the mesh wall with the head fluttering of the silkworm. As sericin still exists with a certain viscosity, the water eventually evaporates and a complete silk fiber is formed. Figure 4 shows the details about silkworm spitting device. Data obtained through the micro-computerized tomography scanning of the structure of the spinneret and the muscles around the mouth suggest that, during the spinning process, the mouth of the silkworm acts as an extrusion mold and applies stress to the gelatinous silk [118]. The volume of the spinning coating contained in the silk gland varies from a few tens of nanoliters to a few microliters. Moreover, the slow spinning speeds of insects such as silkworms may be one of the reasons that they are able to produce high-strength and high-performance fibers, but it is not conducive to industrial production [86].

Hyoung-Joon Jin and David L. Kaplan [119] simulated the self-assembly process of filipin proteins using a mixture of filipin proteins and PEOs, where the PEOs were used in place of sericin in the experiments. It was found that SF formed globular structures due to this confusion. Thus, they found that the SF would pre-form micelles and then assemble them into spherical structures and finally form microfibrillar structures under a physical shear field. By studying the differences between natural spinning and regenerated spinning, Wang et al. [120] concluded that simple extrusion during spinning does not result in regenerated mercerized fibers with mechanical properties similar to those of natural fibers. The cell’s stretching contraction is one of the driving forces that pushes the SF solution out of the silkworm to form the cocoon, while the contraction of the spatula muscle is the switch and driving force that causes the SF solution to be discharged from the body. At the same time, the head traction of the domestic silkworm acts as the third driving force for the expulsion of the SF solution from the body and is an important factor in cocoon molding. Shao et al. [14] found that the mechanical properties of silks artificially withdrawn at a steady rate from mature live silkworms were much better than those of cocoon silk, and silks artificially withdrawn at different rates had different mechanical properties, which, in some conditions, could even be comparable to those of the spider’s main glandular silk. These findings show that it is not only the structure of the amino acid sequence that determines the extraordinary mechanical properties of structured fiber materials such as silk, but also the spinning conditions and the silk–protein aggregation structure, which may play an even more important role. This provides a new direction for research on spinning conditions such as silk dissolution, the regenerated SF stock solution type and concentration, and coagulation baths.

## 4. Bionic Spinning

Currently, the most basic method of artificial spinning is as follows: obtain a solution containing SF, extract the solute via the solvent diffusion method in a specific device, and obtain regenerated silk fibers. The artificial spinning method has been developed over almost 60 years; the recycled fibers obtained have improved in strength and stiffness, but their ductility and toughness are still not comparable to those of natural fibers. Koeppel and Holland [121] summarized the regenerated fibers obtained throughout the 21st century in terms of five dimensions: stiffness, tenacity, diameter, ductility, and strength. They found that all properties of regenerated silk fibers have been improved over time, but the wet-spinning method seems to have reached its limits in terms of strength and tenacity. The emergence of the dry spinning method provides a new opportunity to improve the strength and stiffness of recycled fibers. However, data indicate that high strength and stiffness cannot be combined with high toughness and ductility.

### 4.1. Imitation of Solvents

SF can only be solubilized in water, requiring the addition of other solvents to dissolve it. Some commonly used dissolving methods are acids, alkalis, neutral salts, enzymes, and ionic solutions. Among them, acids, alkalis, and enzymes will decompose the large peptide chains of SF into short peptide chains and amino acids, which will seriously reduce the molecular weight of the SF, affecting its physicochemical properties and its subsequent application.

Nowadays, neutral salts, mixed solutions of salts, and organic compounds are mainly used for dissolution. Table 5 briefly lists the types of solvents commonly chosen.

Acids were first used in an attempt to dissolve SF. As early as 1907 and 1928, Bauman and Diesser used formic acid to dissolve silk fibers, respectively, and this was followed by the use of phosphoric acid [138], sulfuric acid [139], or various acidic salts to dissolve silk fibers. In the 1980s, we used ammonium sulfate and sodium sulfate to dissolve silk fibers and successfully produced regenerated silk fibers via wet spinning with the resulting solution. In 1983, comparing brominated and formalized SF, it was found that the degree of hydrolysis of the three types of SF was in the order of original SF > brominated SF > formalized SF. However, the degree of acidic hydrolysis was difficult to control. Acid hydrolysis and alkali hydrolysis will destroy the complete amino acid chain of SF. For example, acids will destroy the colored amino acids to produce a black insoluble material, while alkali hydrolysis will lead to the racemization of the amino acids into other products. For neutral solutions, enzymatic solubilization can be used. The specificity of enzymes can be used to hydrolyze proteins into specific free amino acids and polypeptide chains, without destroying the original amino acids. Many of the enzymes used for hydrolysis are proteolytic enzymes that come from the human body, such as trypsin, which is not harmful to humans. However, enzymatic solubilization is performed by reducing the molecular weight of SF to achieve solubilization, which degrades the various mechanical properties of the SF and changes the original characteristics. Therefore, the regenerated SF is mostly used in food processing and other fields.

**Figure 5:**
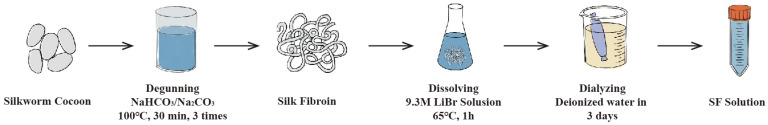
SF dissolved using 9.3 M LiBr aqueous solution as solvent and dialyzed.

The use of neutral salts to dissolve silk pigments is the most common method. The cations that are often chosen include Li^+^, Ca^2+^, Mg^2+^, Zn^2+^, etc., and the anions include Cl^−^, Br^−^, NO_3_^−^, SCN^−^, etc. Figure 5 shows the commom way to dissolve SF by using 9.3 M LiBr as solvent. Moreover, copper ammonium and copper ethylenediamine solutions can be used as solvents for silk fibers [140]. ^13^C NMR was used to study the solution phenomenon of SF in a CaCl_2_ solution. It was found that C=O peaks appeared on the graph; thus, it was surmised that the action of salt ions accelerated the speed of peptide chain movement, weakened the van der Waals forces between the peptide chains, and caused the destruction of the hydrogen bonds. It was also found that salt ions could be exchanged with some elements in the side groups of the peptide chains or react with the residues, further weakening the interactions between the peptide chains and gradually decreasing the crystallinity of SF. On the other hand, salt ions increase the surface charges of SF molecules and enhance the interaction with water molecules. Many comparative experiments have been conducted on the dissolution of SF by different neutral salts, e.g., at a 30–40% concentration, the solubility rates are in the order of CaCl_2_ > MgCl_2_ > ZnCl_2_; however, at a 50–60% concentration, the order is ZnCl_2_ > CaCl_2_ > MgCl_2_. Through comparisons with polar organic solvents such as ethanol, methanol, and acetone and non-polar organic solvents such as N,N-dimethylformamide and dimethyl sulfoxide, it was found that the solubility of SF is susceptible to the polarity of the organic solvent and that polar organic solvents may lead to β-sheets in the SF structure.

In addition to an aqueous solution, SF can also be dissolved in an ionic liquid. Ionic liquids are salts formed from organic cations and organic anions that are liquid at room temperature; hence, they are also known as molten salts. Junghans et al. [141] first discovered that silk and spider silk can undergo solubilization in ionic liquids, providing a new approach to silk proteolysis. When comparing 10 ionic liquids, such as 1-butyl-3-methylimidazole acetate ([Bmim]AC) and 1,3-dimethylimidazole dimethyl phosphate ([Mmim]DMP), it was found that the [Bmim]AC ionic liquid had the best solubility for filipin, and the maximum solubility capacity of filipin proteins reached 15% at 75 °C for 840 min. There are still some issues to be addressed regarding the dissolution of SF using ionic liquids: the melting points of ionic liquids themselves are generally high, and the dissolution process requires an oxygen-free and anhydrous environment, which is challenging to achieve. At the same time, obtaining the SF dissolved in an ionic liquid has emerged as a new challenge.

### 4.2. Imitation of Behavior

**Figure 6:**
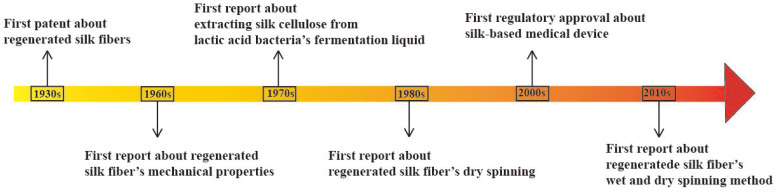
Development of dry and wet spinning for SF.

In the 1930s, research was conducted on the spinning of regenerated silk fibers. By the 1990s, much research had been reported in this area, as well as some progress in spinning using regenerated silk fibers. By analyzing the ways in which the silkworm spins silk naturally, preliminary ideas have been obtained regarding methods to obtain regenerated SF fibers. For example, the spinning solution is extruded from a very thin tube and the solvent in the spinning solution is removed in the atmosphere or in a solution to obtain intact fibers. After nearly 70 years of research and practice, this method has gained recognition. The devices and means of spinning have been continuously optimized in experiments, achieving a significant improvement in the mechanical properties of the regenerated silk protein fibers. Figure 6 gives a brief summary about the development of dry and wet spinning for regenerated silk fiber. Overall, the main methods regarding the regeneration of sericin protein fibers can be classified according to the synthesis method: wet spinning, dry spinning, and wet-and-dry spinning.

Wet spinning involves solidifying and precipitating the spinning solution into a coagulation bath to form primary fibers after the spinning solution is extruded from the spinneret via a screw pump/metering pump. This method of spinning was the earliest documented method used to spin regenerated SF. In a 1933 patent by Esselen, the act of spinning with regenerated silk fibers was mentioned for the first time. From the 1930s to the 1950s, the solvent and coagulation bath systems for the wet spinning of regenerated SF were not fixed, and most of them were in the exploration stage [138]. Some scholars have been inspired by the solvent/coagulation bath system for cellulose fiber spinning [142]. Using a copper hydroxide, ammonia, and sodium hydroxide solution to dissolve SF, and using sodium bisulfite as a coagulation bath for wet spinning, regenerated SF fibers were successfully prepared. At the same time, researchers have been inspired by scientist E.E. Hughes’ man-made animal gelatin fibers, presented in 1866, which were spun using an acidic solvent to dissolve SF. In the 1960s, Yazawa [143] performed wet spinning using a magnesium nitrate solution to dissolve silk, with saturated ammonium sulfate as a coagulation bath. This method obtained regenerated SF fibers with strength of approximately 0.18 GPa and elongation of 20–25%. This may have been the first report on the mechanics of the wet spinning of regenerated SF fibers. The acquisition of these fibers marked a major breakthrough in the field of regenerated SF fibers, but, due to the limitations of the technical level at that time, the diameter of the regenerated SF fibers was generally coarse (>100 μm) [144], being five times that of natural silk fibers [145]. If compared with a single natural SF fiber within natural silk, the difference in diameter reaches 10 times or more. Yan et al. [146] found that changes in calcium ions present in the spinning solution had a positive effect on fiber formation. Meanwhile, M. Andersson’s team examined the pH of the solution in different silk glands and found that the pH of the solution gradually decreased as the silk solution moved from the rear silk glands to the middle and front silk glands [147], providing a new perspective on bionic spinning.

Prior to the beginning of the 21st century, there were further advances in the wet spinning of regenerated SF fibers [148]. Ishizaka et al. [138] dissolved SF in a phosphoric acid solution, using a mixed ammonium sulfate/sodium sulfate solution as a coagulation bath, to obtain primary fibers. The primary fibers were placed in a 90% methanol solution for tensile post-treatment, resulting in fibers with strength of up to 250 MPa. Yao et al. [149] used hexafluoroacetone as a solvent and methanol as a solidifying bath; the strength of the fibers obtained from this treatment reached 180 MPa. Moreover, researchers have reported regenerated SF fibers with fiber strengths of 120 MPa and 273 MPa, respectively, obtained using formic acid [150] and phosphoric acid as solvents and methanol as a solidifying bath. Methanol and ethanol have been considered in several studies as the optimal options for coagulation baths due to their low price, wide availability, and low toxicity. By adjusting the solvent and coagulation bath formulations and the post-treatment conditions, the diameter of the fibers obtained by wet spinning can be significantly reduced and the mechanical properties substantially improved. However, the spinning speed in wet spinning is slow, generally 50–100 m/min; moreover, the wet spinning process is cumbersome, the financial investments required for plant construction and equipment are high, the cost is high, and there is still a certain amount of pollution transmitted to the water environment and the atmosphere. The rapid production of regenerated silk fibers has also become an important goal.

**Figure 7:**
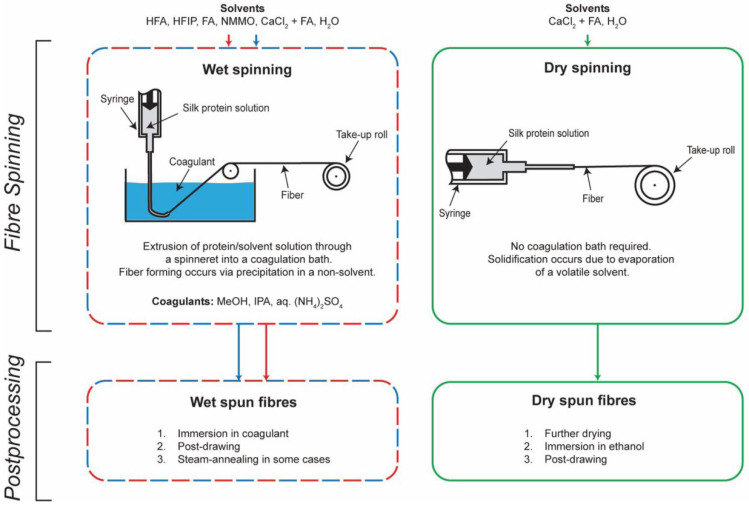
Methods for production of regenerated silk fibers. Copyright © 2017 American Chemical Society, reproduced with permission from [121].

Dry spinning methods for chemical fibers may be able to achieve this goal. This spinning method is highly compatible with the physiological method of silk spitting adopted by silkworms, and it can simulate the mechanisms and conditions of natural spinning. Dry spinning is characterized by the direct contact of the spinning liquid with the air after it is extruded from the spinneret, and the solvent precipitation, curing, and stretching of the SF fibers are completed in the air [151,152,153,154]. The first report on dry spinning was published in 1991 [155]—nearly half a century later compared to wet spinning, but the research is developing rapidly. The regenerated SF fibers obtained by this method are much thinner than those obtained via wet spinning, and they show some enhancements in terms of strength. This may be attributed to the fact that the rapid evaporation of the solvent is able to reduce the formation of internal voids in the fibers. At the same time, it is possible to induce the formation of a β-sheet structure in the SF, which results in the formation of a large number of crystalline areas. Wei et al. [156] spun regenerated SF fibers in the air via dry spinning, after which the fibers were post-stretched by immersing them in an 80% aqueous solution of ethanol to improve the mechanical properties. Through a quantitative analysis, it was found that the ratio of β-sheet and α-helix conformations increased significantly at the beginning of the soaking process and approached stability after 90 min. The regenerated SF fibers showed an increased fracture stress of 301 MPa. However, there was no significant relationship between the pH of the raw material and the mechanical properties of the regenerated fibers. This may be due to the fact that ethanol acts as an undesirable solvent to induce the massive crystallization of SF [157]. In contrast, small changes in pH do not have a significant effect on the structure of the fibers. Figure 7 shows the usual way to make regenerated silk fiber.

By combining the above two methods, we can obtain the wet-and-dry spinning method, which is also known as wet-and-dry spray spinning [158]. When spinning, the spinning liquid extruded from the spinneret first passes through an air layer of a certain height, allowing the gravity drafting of the silk strips in the preliminary stage, which can effectively regulate the process of microstructure formation. This property enables the use of relatively large spinneret apertures when spinning, reducing the limitation on the viscosity of the spinning liquid. Then, the silk strip enters the solidifying bath for curing, followed by pre-stretching, washing, and other processes performed on the primary fibers according to the application, to obtain regenerated SF fibers that meet the specific requirements. Zhang et al. [64] attempted to use in-house-fabricated wet-and-dry spinning equipment. They chose to blend the dialyzed SF solution with polyethylene glycol, and ethanol was used as a coagulation bath to prepare regenerated SF fibers with different SF concentrations. An N-methyl morpholine-N-oxide (NMMO) solution can also be selected as the regenerated silk protein solvent, with methanol as the coagulation bath, to solidify and precipitate the SF and stretch it into fibers. The fibers were post-drafted afterwards, resulting in regenerated silk fibers with strength of 0.31 MPa.

### 4.3. Possibility of Liquid Crystal Spinning

Various artificial spinning methods have been used to produce regenerated silk fibers, but it is still difficult to maintain the advantages of natural silk in terms of the structural hierarchy and mechanical properties [159]. The molecular weight of SF is very high, but the viscosity of the solution is much lower than that of synthetic polymers with comparable or even smaller molecular weights; in addition, the spinning speed and pressure of the domestic silkworm are much lower compared to the synthetic fiber spinning process. Based on the above, some scholars have hypothesized that liquid crystal state structures may exist in liquid SF. In 1989, Li et al. [160] found that the silk-spun liquid exhibited a liquid crystal state, which was both fluid and partially maintained the ordered arrangement of the molecules of the substance, and it exhibited anisotropy in its physical properties. Meanwhile, Magoshi’s team reported that the liquid SF extruded from the anterior silk gland formed a nematic liquid crystal phase [161]. The formation of silk fibers is considered to be based on the mechanical denaturation of liquid SF. Furthermore, the SF solution undergoes shear stress and grows during the concentration process, causing the crystalline solidification of the proteins in the solution. The sericin encapsulated in the peripheries of the SF is considered as a lubricant, ensuring the smooth movement of the solution during the constant squeezing and concentration process. This mechanism provides a relatively simple illustration of the process by which most silk-spinning species produce silk. Martel et al. [80] reported the stress–strain behavior of SF solutions is obtained directly from the silkworm itself. They observed constant non-Newtonian fluids in the test range of 10–1000 mm min^−1^, but a yield point existed at 500 mm min^−1^, at which the test solution became opaque and delamination occurred in the solution. X-ray tests showed that a large number of well-oriented β-sheet structures existed in the solution at this point, while the critical shear rate further increased with the increasing system temperature, which is consistent with the natural spinning of silkworms.

Later, K. Kerkam et al. [161] observed characteristic birefringence in the molecular arrangement within the natural silk glands, while, as the solvent evaporated, natural silk showed nematic liquid crystals. D. P. Knight and Vollrath [162] observed nematic ripples in the spinning fluid in the anterior silk gland of Bombyx mori. sericea under an atomic force microscope. P. Willcox et al. [163] noted the presence of a cholesteric phase in the preductal portion of the major filament-producing gland in the Bombyx mori. and Nephila claviers spiders, speculating that the solutions in the cholesteric phase might be twisted and converted into the nematic state via flow and extrusion. This discovery may provide a plausible explanation for the strong mechanical properties of silkworm and spider silks. Most industrial liquid crystal polymers are thermotropic liquid crystals. The melt spinning of 2-chlorohydroquinone, 1,4-cyclohexanedimethanol, and terephthaloyl chloride with liquid crystal co-polyesters at 120 °C–170 °C. 4’-dihydroxybiphenyl (B), 4-hydroxybenzoic acid (H), and aliphatic groups were able to produce better crystallinity and a higher molecular weight at 190 °C [164]. The natural silk spinning solution may be a solvatochromic liquid crystal, which are more dependent on the solvent induction of SF for crystal formation [165]. Compared to thermotropic liquid crystals, solvatochromic transformations require less energy but larger amounts of solvent. The results obtained by L. D. Rey through the programmed simulation of the liquid crystal spinning of SF were consistent with the phenomena observed in the natural sericin glands [166]. This may provide new ideas for the artificial spinning of silk, i.e., it may be possible to obtain an SF solution with a liquid crystal structure that is suitable for subsequent spinning by adjusting the solvent formulation, the treatment temperature, and the means of treatment.

## 5. Discussion and Conclusions

By summarizing the currently available artificial spinning methods and spinning information, we can observe a number of features that are crucial for fiber shaping and performance improvement.

From a behavioral viewpoint, most spinning methods are carried out by copying the spitting of silk by silkworms. The spinning solution is pressurized using mechanical equipment to pass through tiny diameter spouting holes, and the shear force given by the walls of the holes during the process induces the transformation of the secondary structure of the silk fibroin inside the solution, facilitating silk formation. Wet spinning, as the earliest spinning method to be widely promoted, improves the fineness of the fibers obtained, and the mechanical properties have been substantially enhanced through the continuous improvement of the production process and raw materials. However, the spinning speed in wet spinning is slow, generally 50–100 m/min; in addition, the wet spinning process is complex, the financial investments required in plant construction and equipment are high, the cost is high, and there is still a certain amount of pollution transmitted to the water environment and the atmosphere. Dry spinning can simulate the mechanisms and conditions of silkworm spitting, and the spinning speed and yield are superior to those of wet spinning. However, a large number of volatile solvents are used in the production process, creating stringent requirements for the factory environment. By combining the above two methods, we can obtain the wet-and-dry spinning method. This method combines the advantages of dry spinning and wet spinning, offering more choice regarding the fiber fineness and solution concentration. However, this method has high process requirements and has not yet been popularized.

The SF in natural silk has four secondary structures: random, α-helix, β-sheet, and β-turn structures. They are interconnected, endowing natural fibers with high strength and toughness. However, most of the regenerated SF fibers have a single internal secondary structure, which is essentially a β-sheet structure, and present poor strength and brittleness. At the same time, most recycled fibers use SF as the main body of the fiber, achieving comparable fiber fineness to natural fibers. However, the SF is formed by two SF fibers wrapped by sericin, with the fineness of a single fiber; thus, the required fineness is still far from being reached. One reason that silk fibers perform strongly when used alone is their unique structure consisting of sericin wrapped around SF.

If a similar spinning device is designed based on the cross-sectional structure of silk, the mechanical properties of the fibers obtained might be better improved. By studying the behavior of silkworms during the natural spinning process, it was found that, in addition to the extrusion given by the muscles external to the glands to push the spinning liquid toward the front end, head shaking might contribute to the fiber property enhancement. This is why most artificially spun silks need to be post-drafted to induce recrystallization and enhance the mechanical properties of the fibers.

From the solvent viewpoint, in order to solubilize SF, the bonding connections in the internal crystalline regions need to be broken, which could have an effect on the molecular structure of the SF. The crystalline structure of natural SF is stable, where the ratio of H chain:L chain: P25 = 6:6:1. In contrast, when obtaining SF in its molecular state, the processes of degumming and solubilization cause damage to its structure, and it is impossible to closely examine the changes in the molecular chains within the solution. The acidic solvents used in the previous stage are strongly volatile and play an important role in the rapid formation of fibers, but the destruction of the molecular weight of the SF by these acidic solvents cannot be ignored. For example, the mechanical properties of regenerated SF fibers obtained using formic acid as a solvent showed a continuous decrease over 5 days. Dissolution can be achieved later using salt solutions/organic solvents, but the need to maintain high dissolution temperatures and the control of the temperature during subsequent dialysis need to be further investigated.

Over the past 60 years, researchers have extensively studied the physiology and biochemistry of silk and have obtained regenerated SF fibers that are much improved in a number of areas, including performance and diameter, but clearly there is still considerable room for improvement. We expect that, in the near future, it will be possible to obtain regenerated fibers that are comparable to natural silk fibers, or even superior to natural fibers, in all aspects. We also hope that the ideas presented in this article inspire readers and thus exert a broad and significant impact.

## Figures and Tables

**Table 2:** Amino acid content of different types of SF and spider silk.

Amino Acid	Amino Acid Composition (mol%)						
	*B. Mori*				Serotonin Sericin	Mulberry (Bivoltine)	Mulberry (Crossbreed)
	All	H Chain	L Chain	P25 Glycoprotein Chain			
Aspartic	1.9	0.4	7.4	5.9	8.22	1.64	1.49
Glutamic	1.4	0.6	6.1	3.4	2.12	1.77	1.53
Serine	12.2	12.1	9.8	6.9	12.9	10.38	10.85
Glycine	42.9	45.9	9	4.4	25.85	43.45	43.73
Histidine	0.2	0.1	2.1	5.1	1.56	0.13	0.15
Arginine	0.5	0.3	4.1	5.9	5.13	1.13	1.16
Threonine	0.9	0.9	2.9	5.9	0.47	0.92	0.76
Alanine	30	30.3	13.5	4.4	44.1	27.56	28.36
Proline	0.5	0.3	2.9	5.9	0.47	0.79	0.76
Tyrosine	4.8	5.3	4.1	4.9	9.1	5.58	5.76
Valine	2.5	1.8	7	4.4	1.5	2.37	2.89
Methionine	0.1	0.1	0.4	0.5	0.5	0.19	0.11
Cystine	0.1	0.1	1.2	3.9	0.23	0.13	0.12
Lsoleusine	0.2	0.2	8.2	6.9	0.38	0.75	0.78
Leucine	0.6	0.1	7	9.4	0.4	0.73	0.75
Phenylalanine	0.6	0.6	2.9	7.4	0.47	0.14	0.18
Tryptophan	0.2	0.7	9.8	11.4	1.75	0.73	0.75
Lysine	0.4	0.2	1.6	3.4	0.21	0.23	0.25
Refs.	[30,31]	[32,33,34]			[35]	[34]	

**Table 3:** Interval distribution of secondary structures of SF analyzed via FITR.

Assignment	Amide I	Amide II	Amide III	Amide IV
α-helix	1656–1662	1530–1540	1266	669
β-sheet	1697–1703 1620–1635	1525–1530	1260–1265	900
β-turn	1663–1668	1520–1535	1235	650
Random	1647–1655	1534–1548	1240–1230	/

**Table 4:** Interval distribution of secondary structures of SF analyzed via Raman.

Assignment	Amide I	Amide III	C-C
α-helix	1645–1658	1264–1310	1103–1108
β-sheet	1665–1680	1220–1245	1020–1060, 1185
Random	1660–1665	1250	<1106

**Table 5:** Common solvents for SF.

Solvent	Ratio of Solvent	Temp. (°C)	Refs.
Calcium chloride/Formic acid	/	25	[122]
Hydroxyapatite/ Formic acid	Hydroxyapatite content ≤ 5%	25	[123]
Formic acid	98%	25	[124]
Protease XIV	≥3.5 units per mg protein	25	[125]
Proteinase K	≥30 units per mg protein	25	[126]
Papain (lysosomal-like enzyme)	≥10 units per mg protein	25	[127]
Calcium chloride/ Water	5 M	80	[128]
Calcium chloride/Ethanol/Water	1:4:2	80	[129]
Lithium bromide/Water	9.2–9.5 M	60	[130,131,132]
Lithium thiocyanide/Water	9 M	25	[133]
Lithium chloride/DMSO	1 M lithium chloride dissolved in DMSO	60	[134]
[AMIM]Cl	98%	70	[135]
[BMIM]Cl	98%	100	[136]
[BMIM]Br	95%	100	[137]

## Data Availability

Data will be made available on request (due to privacy).

## References

[1] Trossmann V.T., Scheibel T. (2023). Design of Recombinant Spider Silk Proteins for Cell Type Specific Binding. Adv. Healthc. Mater..

[2] Wang Q., McArdle P., Wang S.L., Wilmington R.L., Xing Z., Greenwood A., Cotten M.L., Qazilbash M.M., Schniepp H.C. (2022). Protein Secondary Structure in Spider Silk Nanofibrils. Nat. Commun..

[3] Vendrely C., Scheibel T. (2007). Biotechnological Production of Spider-Silk Proteins Enables New Applications. Macromol. Biosci..

[4] Li J., Li S., Huang J., Khan A.Q., An B., Zhou X., Liu Z., Zhu M. (2022). Spider Silk-Inspired Artificial Fibers. Adv. Sci..

[5] Vollrath F., Porter D. (2006). Spider Silk as Archetypal Protein Elastomer. Soft Matter.

[6] Jokisch S., Scheibel T. (2017). Spider Silk Foam Coating of Fabric. Pure Appl. Chem..

[7] Zhang X., Xia L., Day B.A., Harris T.I., Oliveira P., Knittel C., Licon A.L., Gong C., Dion G., Lewis R.V. (2019). CRISPR/Cas9 Initiated Transgenic Silkworms as a Natural Spinner of Spider Silk. Biomacromolecules.

[8] Zhang H., Zhou F., Jiang X., Cao M., Wang S., Zou H., Cao Y., Xian M., Liu H. (2016). Microbial Production of Amino Acid-Modified Spider Dragline Silk Protein with Intensively Improved Mechanical Properties. Prep. Biochem. Biotechnol..

[9] Wang M.-Y., Zhang J.-P., Chen S.-L., Qi B., Yao X.-Y., Zhang X.-H., Li Y.-T., Yang Z.-H. (2023). Dry-Spinning of Artificial Spider Silk Ribbons from Regenerated Natural Spidroin in an Organic Medium. Macromol. Rapid Commun..

[10] Wu R., Bae J., Jeon H., Kim T. (2022). Spider-Inspired Regenerated Silk Fibroin Fiber Actuator via Microfluidic Spinning. Chem. Eng. J..

[11] Huemmerich D., Scheibel T., Vollrath F., Cohen S., Gat U., Ittah S. (2004). Novel Assembly Properties of Recombinant Spider Dragline Silk Proteins. Curr. Biol..

[12] Fahnestock S.R., Bedzyk L.A. (1997). Production of Synthetic Spider Dragline Silk Protein in Pichia Pastoris. Appl. Microbiol. Biotechnol..

[13] Sirichaisit J., Brookes V.L., Young R.J., Vollrath F. (2003). Analysis of Structure/Property Relationships in Silkworm (*Bombyx mori*) and Spider Dragline (*Nephila edulis*) Silks Using Raman Spectroscopy. Biomacromolecules.

[14] Shao Z., Vollrath F. (2002). Surprising Strength of Silkworm Silk. Nature.

[15] Gosline J.M., Guerette P.A., Ortlepp C.S., Savage K.N. (1999). The Mechanical Design of Spider Silks: From Fibroin Sequence to Mechanical Function. J. Exp. Biol..

[16] Ko F., Jovicic J. (2004). Modeling of Mechanical Properties and Structural Design of Spider Web. Biomacromolecules.

[17] Hermanson K.D., Huemmerich D., Scheibel T., Bausch A.R. (2007). Engineered Microcapsules Fabricated from Reconstituted Spider Silk. Adv. Mater..

[18] Cunniff P.M., Fossey S.A., Auerbach M.A., Song J.W., Kaplan D.L., Adams W.W., Eby R.K., Mahoney D., Vezie D.L. (1994). Mechanical and Thermal Properties of Dragline Silk from the Spider Nephila Clavipes. Polym. Adv. Technol..

[19] Knight D.P., Knight M.M., Vollrath F. (2000). Beta Transition and Stress-Induced Phase Separation in the Spinning of Spider Dragline Silk. Int. J. Biol. Macromol..

[20] Römer L., Scheibel T. (2008). The Elaborate Structure of Spider Silk. Prion.

[21] Kumar S., Doshi H., Srinivasarao M., Park J.O., Schiraldi D.A. (2002). Fibers from Polypropylene/Nano Carbon Fiber Composites. Polymer.

[22] Fedič R., Žurovec M., Sehnal F. (2003). Correlation between Fibroin Amino Acid Sequence and Physical Silk Properties. J. Biol. Chem..

[23] Takasu Y., Yamada N., Kojima K., Iga M., Yukuhiro F., Iizuka T., Yoshioka T. (2023). Fibroin Heavy Chain Gene Replacement with a Highly Ordered Synthetic Repeat Sequence in *Bombyx mori*. Insect Biochem. Mol. Biol..

[24] Sahoo J.K., Hasturk O., Falcucci T., Kaplan D.L. (2023). Silk Chemistry and Biomedical Material Designs. Nat. Rev. Chem..

[25] Ma Y., Luo Q., Ou Y., Tang Y., Zeng W., Wang H., Hu J., Xu H. (2021). New Insights into the Proteins Interacting with the Promoters of Silkworm Fibroin Genes. Sci. Rep..

[26] Indrakumar S., Joshi A., Dash T.K., Mishra V., Tandon B., Chatterjee K. (2023). Photopolymerized Silk Fibroin Gel for Advanced Burn Wound Care. Int. J. Biol. Macromol..

[27] Kostag M., Jedvert K., El Seoud O.A. (2021). Engineering of Sustainable Biomaterial Composites from Cellulose and Silk Fibroin: Fundamentals and Applications. Int. J. Biol. Macromol..

[28] De Giorgio G., Matera B., Vurro D., Manfredi E., Galstyan V., Tarabella G., Ghezzi B., D’Angelo P. (2024). Silk Fibroin Materials: Biomedical Applications and Perspectives. Bioengineering.

[29] Park W., Yoon T., Chang H., You J., Na S. (2024). An Atomistic Scale Simulation Study of Structural Properties in the Silk–Fibrohexamerin Complex. Nanoscale.

[30] Fu C., Shao Z., Fritz V. (2009). Animal Silks: Their Structures, Properties and Artificial Production. Chem. Commun..

[31] Zhang X., Berghe I.V., Wyeth P. (2011). Heat and Moisture Promoted Deterioration of Raw Silk Estimated by Amino Acid Analysis. J. Cult. Herit..

[32] Hozumi T. (1981). Properties of Amino Acid Composition of the Tryptic Fragments of the Heavy Chain of Myosin Subfragment-1. J. Biochem..

[33] Ahmad R., Kamra A., Hasnain S.E. (2004). Fibroin Silk Proteins from the Nonmulberry Silkworm Philosamia Ricini Are Biochemically and Immunochemically Distinct from Those of the Mulberry Silkworm *Bombyx mori*. DNA Cell Biol..

[34] Sen K., Babu K.M. (2004). Studies on Indian Silk. I. Macrocharacterization and Analysis of Amino Acid Composition. J. Appl. Polym. Sci..

[35] Shao Z., Vollrath F., Yang Y., Thøgersen H.C. (2003). Structure and Behavior of Regenerated Spider Silk. Macromolecules.

[36] Omenetto F.G., Kaplan D.L. (2010). New Opportunities for an Ancient Material. Science.

[37] Wöltje M., Isenberg K.L., Cherif C., Aibibu D. (2023). Continuous Wet Spinning of Regenerated Silk Fibers from Spinning Dopes Containing 4% Fibroin Protein. Int. J. Mol. Sci..

[38] Lucas F., Shaw J.T.B., Smith S.G. (1957). The Amino Acid Sequence in a Fraction of the Fibroin of *Bombyx mori*. Biochem. J..

[39] Zhang K., Si F.W., Duan H.L., Wang J. (2010). Microstructures and Mechanical Properties of Silks of Silkworm and Honeybee. Acta Biomater..

[40] Lucas F., Shaw J.T.B., Smith S.G. (1962). Some Amino Acid Sequences in the Amorphous Fraction of the Fibroin of *Bombyx mori*. Biochem. J..

[41] Nadiger G.S., Bhat N.V., Padhye M.R. (1985). Investigation of Amino Acid Composition in the Crystalline Region of Silk Fibroin. J. Appl. Polym. Sci..

[42] Yamaguchi K., Kikuchi Y., Takagi T., Kikuchi A., Oyama F., Shimura K., Mizuno S. (1989). Primary Structure of the Silk Fibroin Light Chain Determined by cDNA Sequencing and Peptide Analysis. J. Mol. Biol..

[43] Jiang F., Liu K., Zhao M., Tao X., Hu X., Lu S. (2020). Tunable High-Molecular-Weight Silk Fibroin Polypeptide Materials: Fabrication and Self-Assembly Mechanism. ACS Appl. Bio Mater..

[44] Eliaz D., Paul S., Benyamin D., Cernescu A., Cohen S.R., Rosenhek-Goldian I., Brookstein O., Miali M.E., Solomonov A., Greenblatt M. (2022). Micro and Nano-Scale Compartments Guide the Structural Transition of Silk Protein Monomers into Silk Fibers. Nat. Commun..

[45] Zafar M.S., Belton D.J., Hanby B., Kaplan D.L., Perry C.C. (2015). Functional Material Features of *Bombyx mori* Silk Light versus Heavy Chain Proteins. Biomacromolecules.

[46] Kojima K., Kuwana Y., Sezutsu H., Kobayashi I., Uchino K., Tamura T., Tamada Y. (2007). A New Method for the Modification of Fibroin Heavy Chain Protein in the Transgenic Silkworm. Biosci. Biotechnol. Biochem..

[47] Long D., Lu W., Zhang Y., Guo Q., Xiang Z., Zhao A. (2015). New Insight into the Mechanism Underlying Fibroin Secretion in Silkworm, *Bombyx mori*. FEBS J..

[48] Pérez-Rigueiro J., Elices M., Llorca J., Viney C. (2001). Tensile Properties of Silkworm Silk Obtained by Forced Silking. J. Appl. Polym. Sci..

[49] Asakura T., Ohgo K., Ishida T., Taddei P., Monti P., Kishore R. (2005). Possible Implications of Serine and Tyrosine Residues and Intermolecular Interactions on the Appearance of Silk I Structure of *Bombyx mori* Silk Fibroin-Derived Synthetic Peptides:  High-Resolution 13C Cross-Polarization/Magic-Angle Spinning NMR Study. Biomacromolecules.

[50] Zhou C.-Z., Confalonieri F., Jacquet M., Perasso R., Li Z.-G., Janin J. (2001). Silk Fibroin: Structural Implications of a Remarkable Amino Acid Sequence. Proteins Struct. Funct. Bioinform..

[51] Murphy A.R., Kaplan D.L. (2009). Biomedical Applications of Chemically-Modified Silk Fibroin. J. Mater. Chem..

[52] Ha S.W., Tonelli A.E., Hudson S.M. (2005). Structural Studies of *Bombyx mori* Silk Fibroin during Regeneration from Solutions and Wet Fiber Spinning. Biomacromolecules.

[53] Takahashi Y., Gehoh M., Yuzuriha K. (1991). Crystal Structure of Silk (*Bombyx mori*). J. Polym. Sci. Part B Polym. Phys..

[54] Asakura T., Ogawa T., Naito A., Williamson M.P. (2020). Chain-Folded Lamellar Structure and Dynamics of the Crystalline Fraction of *Bombyx mori* Silk Fibroin and of (Ala-Gly-Ser-Gly-Ala-Gly) Model Peptidesn. Int. J. Biol. Macromol..

[55] Valluzzi R., Gido S.P. (1997). The Crystal Structure of *Bombyx mori* Silk Fibroin at the Air–Water Interface. Biopolymers.

[56] Sarkar A., Connor A.J., Koffas M., Zha R.H. (2019). Chemical Synthesis of Silk-Mimetic Polymers. Materials.

[57] Taketani I., Nakayama S., Nagare S., Senna M. (2005). The Secondary Structure Control of Silk Fibroin Thin Films by Post Treatment. Appl. Surf. Sci..

[58] Mu X., Amouzandeh R., Vogts H., Luallen E., Arzani M. (2023). A Brief Review on the Mechanisms and Approaches of Silk Spinning-Inspired Biofabrication. Front. Bioeng. Biotechnol..

[59] Tanaka K., Kajiyama N., Ishikura K., Waga S., Kikuchi A., Ohtomo K., Takagi T., Mizuno S. (1999). Determination of the Site of Disulfide Linkage between Heavy and Light Chains of Silk Fibroin Produced by *Bombyx mori*. Biochim. Biophys. Acta Protein Struct. Mol. Enzymol..

[60] Marsh R.E., Corey R.B., Pauling L. (1955). An Investigation of the Structure of Silk Fibroin. Biochim. Et Biophys. Acta.

[61] Tanaka K., Inoue S., Mizuno S. (1999). Hydrophobic Interaction of P25, Containing Asn-Linked Oligosaccharide Chains, with the H-L Complex of Silk Fibroin Produced by *Bombyx mori*. Insect Biochem. Mol. Biol..

[62] Zhou C.-Z., Confalonieri F., Medina N., Zivanovic Y., Esnault C., Yang T., Jacquet M., Janin J., Duguet M., Perasso R. (2000). Fine Organization of *Bombyx mori* Fibroin Heavy Chain Gene. Nucleic Acids Res..

[63] Ambrose E.J., Bamford C.H., Elliott A., Hanby W.E. (1951). Water-Soluble Silk: An α-Protein. Nature.

[64] Zhang X., Pan Z. (2019). Microstructure Transitions and Dry-Wet Spinnability of Silk Fibroin Protein from Waste Silk Quilt. Polymers.

[65] Kratky O., Schauenstein E. (1951). X-Ray and U.-V. Spectrographic Investigations of Fibrous and Globular Modifications of Silk Fibroin. Discuss. Faraday Soc..

[66] Koga N., Tatsumi-Koga R., Liu G., Xiao R., Acton T.B., Montelione G.T., Baker D. (2012). Principles for Designing Ideal Protein Structures. Nature.

[67] Rousseau M.-E., Beaulieu L., Lefèvre T., Paradis J., Asakura T., Pézolet M. (2006). Characterization by Raman Microspectroscopy of the Strain-Induced Conformational Transition in Fibroin Fibers from the Silkworm Samia Cynthia Ricini. Biomacromolecules.

[68] Drummy L.F., Phillips D.M., Stone M.O., Farmer B.L., Naik R.R. (2005). Thermally Induced α-Helix to β-Sheet Transition in Regenerated Silk Fibers and Films. Biomacromolecules.

[69] Zhou Y., Tang R.-C. (2017). Natural Flavonoid-Functionalized Silk Fiber Presenting Antibacterial, Antioxidant, and UV Protection Performance. ACS Sustain. Chem. Eng..

[70] Koh L.-D., Cheng Y., Teng C.-P., Khin Y.-W., Loh X.-J., Tee S.-Y., Low M., Ye E., Yu H.-D., Zhang Y.-W. (2015). Structures, Mechanical Properties and Applications of Silk Fibroin Materials. Prog. Polym. Sci..

[71] Fossey S.A., Némethy G., Gibson K.D., Scheraga H.A. (1991). Conformational Energy Studies of β-Sheets of Model Silk Fibroin Peptides. I. Sheets of Poly(Ala-Gly) Chains. Biopolymers.

[72] Cheng K., Tao X., Qi Z., Yin Z., Kundu S.C., Lu S. (2021). Highly Absorbent Silk Fibroin Protein Xerogel. ACS Biomater. Sci. Eng..

[73] Yoshioka T., Kameda T., Tashiro K., Ohta N., Schaper A.K. (2017). Transformation of Coiled α-Helices into Cross-β-Sheets Superstructure. Biomacromolecules.

[74] Athiyarath V., Madhusudhanan M.C., Kunnikuruvan S., Sureshan K.M. (2022). Secondary Structure Tuning of a Pseudoprotein Between β-Meander and α-Helical Forms in the Solid-State. Angew. Chem. Int. Ed..

[75] Lazo N.D., Downing D.T. (1999). Crystalline Regions of *Bombyx mori* Silk Fibroin May Exhibit β-Turn and β-Helix Conformations. Macromolecules.

[76] Asakura T., Watanabe Y., Itoh T. (1984). NMR of Silk Fibroin. 3. Assignment of Carbonyl Carbon Resonances and Their Dependence on Sequence and Conformation in *Bombyx mori* Silk Fibroin Using Selective Isotopic Labeling. Macromolecules.

[77] Jiang T., Zhou P., Jiang T., Zhou P. (2011). Environment-Induced Silk Fibroin Conformation Based on the Magnetic Resonance Spectroscopy. On Biomimetics.

[78] Asakura T., Okushita K., Williamson M.P. (2015). Analysis of the Structure of *Bombyx mori* Silk Fibroin by NMR. Macromolecules.

[79] Lotz B., Keith H.D. (1971). The Crystal Structures of Poly(LAla-Gly-Gly-Gly)II and Poly(LAla-Gly-Gly)II. J. Mol. Biol..

[80] Martel A., Burghammer M., Davies R.J., Riekel C. (2007). Thermal Behavior of *Bombyx mori* Silk: Evolution of Crystalline Parameters, Molecular Structure, and Mechanical Properties. Biomacromolecules.

[81] Inouye H., Fraser P.E., Kirschner D.A. (1993). Structure of Beta-Crystallite Assemblies Formed by Alzheimer Beta-Amyloid Protein Analogues: Analysis by x-Ray Diffraction. Biophys. J..

[82] Saitoh H., Ohshima K., Tsubouchi K., Takasu Y., Yamada H. (2004). X-Ray Structural Study of Noncrystalline Regenerated *Bombyx mori* Silk Fibroin. Int. J. Biol. Macromol..

[83] Lu Q., Huang Y., Li M., Zuo B., Lu S., Wang J., Zhu H., Kaplan D.L. (2011). Silk Fibroin Electrogelation Mechanisms. Acta Biomater..

[84] Wilson D., Valluzzi R., Kaplan D. (2000). Conformational Transitions in Model Silk Peptides. Biophys. J..

[85] Rousseau M.-E., Lefèvre T., Beaulieu L., Asakura T., Pézolet M. (2004). Study of Protein Conformation and Orientation in Silkworm and Spider Silk Fibers Using Raman Microspectroscopy. Biomacromolecules.

[86] Lefèvre T., Paquet-Mercier F., Rioux-Dubé J.-F., Pézolet M. (2012). Structure of Silk by Raman Spectromicroscopy: From the Spinning Glands to the Fibers. Biopolymers.

[87] Goormaghtigh E., Cabiaux V., Ruysschaert J.-M. (1990). Secondary Structure and Dosage of Soluble and Membrane Proteins by Attenuated Total Reflection Fourier-Transform Infrared Spectroscopy on Hydrated Films. Eur. J. Biochem..

[88] Kong J., Yu S. (2007). Fourier Transform Infrared Spectroscopic Analysis of Protein Secondary Structures. Acta Biochim. Biophys. Sin..

[89] Jung C. (2000). Insight into Protein Structure and Protein–Ligand Recognition by Fourier Transform Infrared Spectroscopy. J. Mol. Recognit..

[90] Mester L., Govyadinov A.A., Chen S., Goikoetxea M., Hillenbrand R. (2020). Subsurface Chemical Nanoidentification by Nano-FTIR Spectroscopy. Nat. Commun..

[91] Dazzi A., Prater C.B. (2017). AFM-IR: Technology and Applications in Nanoscale Infrared Spectroscopy and Chemical Imaging. Chem. Rev..

[92] Lansford J.L., Vlachos D.G. (2020). Infrared Spectroscopy Data- and Physics-Driven Machine Learning for Characterizing Surface Microstructure of Complex Materials. Nat. Commun..

[93] Hutařová Vařeková I., Hutař J., Midlik A., Horský V., Hladká E., Svobodová R., Berka K. (2021). 2DProts: Database of Family-Wide Protein Secondary Structure Diagrams. Bioinformatics.

[94] Barth A. (2000). The Infrared Absorption of Amino Acid Side Chains. Prog. Biophys. Mol. Biol..

[95] Zheng J.H., Shao J.Z., Liu J.Q. (2002). Studies on Distribution of Amino Acids in Silk Fibroi. Acta Polym. Sin..

[96] Lefèvre T., Rousseau M.-E., Pézolet M. (2007). Protein Secondary Structure and Orientation in Silk as Revealed by Raman Spectromicroscopy. Biophys. J..

[97] Zhong J., Liu Y., Ren J., Tang Y., Qi Z., Zhou X., Chen X., Shao Z., Chen M., Kaplan D.L. (2019). Understanding Secondary Structures of Silk Materials via Micro- and Nano-Infrared Spectroscopies. ACS Biomater. Sci. Eng..

[98] Paquet-Mercier F., Lefèvre T., Auger M., Pézolet M. (2013). Evidence by Infrared Spectroscopy of the Presence of Two Types of β-Sheets in Major Ampullate Spider Silk and Silkworm Silk. Soft Matter.

[99] Koperska M.A., Pawcenis D., Bagniuk J., Zaitz M.M., Missori M., Łojewski T., Łojewska J. (2014). Degradation Markers of Fibroin in Silk through Infrared Spectroscopy. Polym. Degrad. Stab..

[100] Hu X., Kaplan D., Cebe P. (2008). Dynamic Protein−Water Relationships during β-Sheet Formation. Macromolecules.

[101] Xie F., Zhang H., Shao H., Hu X. (2006). Effect of Shearing on Formation of Silk Fibers from Regenerated *Bombyx mori* Silk Fibroin Aqueous Solution. Int. J. Biol. Macromol..

[102] Stair P.C. (2001). Advances in Raman Spectroscopy Methods for Catalysis Research. Curr. Opin. Solid. State Mater. Sci..

[103] Ji D., Deng Y.-B., Zhou P. (2009). Folding Process of Silk Fibroin Induced by Ferric and Ferrous Ions. J. Mol. Struct..

[104] Monti P., Taddei P., Freddi G., Asakura T., Tsukada M. (2001). Raman Spectroscopic Characterization of *Bombyx mori* Silk Fibroin: Raman Spectrum of Silk I. J. Raman Spectrosc..

[105] Monti P., Freddi G., Bertoluzza A., Kasai N., Tsukada M. (1998). Raman Spectroscopic Studies of Silk Fibroin from *Bombyx mori*. J. Raman Spectrosc..

[106] Takahashi Y., Gehoh M., Yuzuriha K. (1999). Structure Refinement and Diffuse Streak Scattering of Silk (*Bombyx mori*). Int. J. Biol. Macromol..

[107] Asakura T., Naito A. (2022). Structure of Silk I (*Bombyx mori* Silk Fibroin before Spinning) in the Dry and Hydrated States Studied Using 13C Solid-State NMR Spectroscopy. Int. J. Biol. Macromol..

[108] Valluzzi R., Gido S.P., Muller W., Kaplan D.L. (1999). Orientation of Silk III at the Air-Water Interface. Int. J. Biol. Macromol..

[109] Lotz B., Keith H.D. (1971). Crystal Structure of Poly(L-Ala-Gly)II: A Model for Silk I. J. Mol. Biol..

[110] Konishi T., Kurokawa M. (1968). The Structure of Silk Fibroin-α. Sen-I Gakkaishi.

[111] Asakura T., Ashida J., Yamane T., Kameda T., Nakazawa Y., Ohgo K., Komatsu K. (2001). A Repeated β-Turn Structure in Poly(Ala-Gly) as a Model for Silk I of *Bombyx mori* Silk Fibroin Studied with Two-Dimensional Spin-Diffusion NMR under off Magic Angle Spinning and Rotational Echo Double Resonance. J. Mol. Biol..

[112] Landau E.M., Popovitz-biro R., Levanon M., Leiserowitz L., Lahav M., Sagiv J. (1986). Langmuir Monolayers Designed for the Oriented Growth of Glycine and Sodium Chloride Crystals at Air/Water Interfaces. Mol. Cryst. Liq. Cryst..

[113] Termonia Y. (1994). Molecular Modeling of Spider Silk Elasticity. Macromolecules.

[114] Vollrath F., Porter D. (2009). Silks as Ancient Models for Modern Polymers. Polymer.

[115] Porter D., Vollrath F. (2008). The Role of Kinetics of Water and Amide Bonding in Protein Stability. Soft Matter.

[116] Porter D., Vollrath F., Shao Z. (2005). Predicting the Mechanical Properties of Spider Silk as a Model Nanostructured Polymer. Eur. Phys. J. E.

[117] Asakura T., Ohgo K., Komatsu K., Kanenari M., Okuyama K. (2005). Refinement of Repeated β-Turn Structure for Silk I Conformation of *Bombyx mori* Silk Fibroin Using 13C Solid-State NMR and X-Ray Diffraction Methods. Macromolecules.

[118] Guo N., Lu K., Cheng L., Li Z., Wu C., Liu Z., Liang S., Chen S., Chen W., Jiang C. (2019). Structure Analysis of the Spinneret from *Bombyx mori* and Its Influence on Silk Qualities. Int. J. Biol. Macromol..

[119] Jin H.-J., Kaplan D.L. (2003). Mechanism of Silk Processing in Insects and Spiders. Nature.

[120] Wang X., Ye X., Guo J., Dai X., Yu S., Zhong B. (2024). Modeling the 3-Dimensional Structure of the Silkworm’s Spinning Apparatus in Silk Production. Acta Biomater..

[121] Koeppel A., Holland C. (2017). Progress and Trends in Artificial Silk Spinning: A Systematic Review. ACS Biomater. Sci. Eng..

[122] Zhang F., You X., Dou H., Liu Z., Zuo B., Zhang X. (2015). Facile Fabrication of Robust Silk Nanofibril Films via Direct Dissolution of Silk in CaCl2–Formic Acid Solution. ACS Appl. Mater. Interfaces.

[123] Ming J., Liu Z., Bie S., Zhang F., Zuo B. (2014). Novel Silk Fibroin Films Prepared by Formic Acid/Hydroxyapatite Dissolution Method. Mater. Sci. Eng. C.

[124] Earland C., Raven D.J. (1954). A New Solvent for Silk. Nature.

[125] Brown J., Lu C.-L., Coburn J., Kaplan D.L. (2015). Impact of Silk Biomaterial Structure on Proteolysis. Acta Biomater..

[126] Altman G.H., Diaz F., Jakuba C., Calabro T., Horan R.L., Chen J., Lu H., Richmond J., Kaplan D.L. (2003). Silk-Based Biomaterials. Biomaterials.

[127] Wongpinyochit T., Johnston B.F., Seib F.P. (2018). Degradation Behavior of Silk Nanoparticles—Enzyme Responsiveness. ACS Biomater. Sci. Eng..

[128] Shen T., Wang T., Cheng G., Huang L., Chen L., Wu D. (2018). Dissolution Behavior of Silk Fibroin in a Low Concentration CaCl_2_-Methanol Solvent: From Morphology to Nanostructure. Int. J. Biol. Macromol..

[129] Gobin A.S., Froude V.E., Mathur A.B. (2005). Structural and Mechanical Characteristics of Silk Fibroin and Chitosan Blend Scaffolds for Tissue Regeneration. J. Biomed. Mater. Res. Part. A.

[130] Yang S., Zhao C., Yang Y., Ren J., Ling S. (2023). The Fractal Network Structure of Silk Fibroin Molecules and Its Effect on Spinning of Silkworm Silk. ACS Nano.

[131] Yin J., Chen E., Porter D., Shao Z. (2010). Enhancing the Toughness of Regenerated Silk Fibroin Film through Uniaxial Extension. Biomacromolecules.

[132] Hirlekar S., Ray D., Aswal V.K., Prabhune A., Nisal A., Ravindranathan S. (2019). Silk Fibroin–Sodium Dodecyl Sulfate Gelation: Molecular, Structural, and Rheological Insights. Langmuir.

[133] Zhao Y., Zhu Z.S., Guan J., Wu S.J. (2021). Processing, Mechanical Properties and Bio-Applications of Silk Fibroin-Based High-Strength Hydrogels. Acta Biomater..

[134] Bucciarelli A., Pal R.K., Maniglio D., Quaranta A., Mulloni V., Motta A., Yadavalli V.K. (2017). Fabrication of Nanoscale Patternable Films of Silk Fibroin Using Benign Solvents. Macromol. Mater. Eng..

[135] Stanton J., Xue Y., Pandher P., Malek L., Brown T., Hu X., Salas-de la Cruz D. (2018). Impact of Ionic Liquid Type on the Structure, Morphology and Properties of Silk-Cellulose Biocomposite Materials. Int. J. Biol. Macromol..

[136] Liu X., Zhang C., Xu W., Liu H., Ouyang C. (2011). Blend Films of Silk Fibroin and Water-Insoluble Polyurethane Prepared from an Ionic Liquid. Mater. Lett..

[137] Phillips D.M., Drummy L.F., Conrady D.G., Fox D.M., Naik R.R., Stone M.O., Trulove P.C., De Long H.C., Mantz R.A. (2004). Dissolution and Regeneration of *Bombyx mori* Silk Fibroin Using Ionic Liquids. J. Am. Chem. Soc..

[138] Ishizaka H., Watanabe Y., ishida KAnd fukumoto O. (1989). Regenerated silk prepeared from ortho phosphoric acid solution of fibroin. J. Sericultural Sci. Jpn..

[139] Kim D.-K., Kim H.-S. (2005). Structure and Characteristic of Chitosan/*Bombyx mori* Silk Fibroin Blend Filems. Polymer.

[140] Chen X., Knight D.P., Shao Z., Vollrath F. (2001). Regenerated Bombyx Silk Solutions Studied with Rheometry and FTIR. Polymer.

[141] Junghans F., Morawietz M., Conrad U., Scheibel T., Heilmann A., Spohn U. (2006). Preparation and Mechanical Properties of Layers Made of Recombinant Spider Silk Proteins and Silk from Silk Worm. Appl. Phys. A.

[142] Freddi G., Pessina G., Tsukada M. (1999). Swelling and Dissolution of Silk Fibroin (*Bombyx mori*) in N-Methyl Morpholine N-Oxide. Int. J. Biol. Macromol..

[143] Madurga R., Gañán-Calvo A.M., Mariscal T., Plaza G.R., Guinea G.V., Elices M., Pérez-Rigueiro J. (2019). Production of Regenerated Silkworm Silk Fibers from Aqueous Dopes through Straining Flow Spinning. Text. Res. J..

[144] Porter D., Guan J., Vollrath F. (2013). Spider Silk: Super Material or Thin Fibre?. Adv. Mater..

[145] Mortimer B., Drodge D.R., Dragnevski K.I., Siviour C.R., Holland C. (2013). In Situ Tensile Tests of Single Silk Fibres in an Environmental Scanning Electron Microscope (ESEM). J. Mater. Sci..

[146] Yan J., Zhou G., Knight D.P., Shao Z., Chen X. (2010). Wet-Spinning of Regenerated Silk Fiber from Aqueous Silk Fibroin Solution: Discussion of Spinning Parameters. Biomacromolecules.

[147] Domigan L.J., Andersson M., Alberti K.A., Chesler M., Xu Q., Johansson J., Rising A., Kaplan D.L. (2015). Carbonic Anhydrase Generates a pH Gradient in *Bombyx mori* Silk Glands. Insect Biochem. Mol. Biol..

[148] Zhang F., Lu Q., Yue X., Zuo B., Qin M., Li F., Kaplan D.L., Zhang X. (2015). Regeneration of High-Quality Silk Fibroin Fiber by Wet Spinning from CaCl2–Formic Acid Solvent. Acta Biomater..

[149] Yao J., Masuda H., Zhao C., Asakura T. (2002). Artificial Spinning and Characterization of Silk Fiber from *Bombyx m Ori* Silk Fibroin in Hexafluoroacetone Hydrate. Macromolecules.

[150] Ki C.S., Lee K.H., Baek D.H., Hattori M., Um I.C., Ihm D.W., Park Y.H. (2007). Dissolution and Wet Spinning of Silk Fibroin Using Phosphoric Acid/Formic Acid Mixture Solvent System. J. Appl. Polym. Sci..

[151] Um I.C., Kweon H., Lee K.G., Ihm D.W., Lee J.-H., Park Y.H. (2004). Wet Spinning of Silk Polymer: I. Effect of Coagulation Conditions on the Morphological Feature of Filament. Int. J. Biol. Macromol..

[152] Arafat M.T., Tronci G., Yin J., Wood D.J., Russell S.J. (2015). Biomimetic Wet-Stable Fibres via Wet Spinning and Diacid-Based Crosslinking of Collagen Triple Helices. Polymer.

[153] Li G., Li Y., Chen G., He J., Han Y., Wang X., Kaplan D.L. (2015). Silk-Based Biomaterials in Biomedical Textiles and Fiber-Based Implants. Adv. Healthc. Mater..

[154] Qiu W., Teng W., Cappello J., Wu X. (2009). Wet-Spinning of Recombinant Silk-Elastin-Like Protein Polymer Fibers with High Tensile Strength and High Deformability. Biomacromolecules.

[155] Wei W., Zhang Y., Zhao Y., Luo J., Shao H., Hu X. (2011). Bio-Inspired Capillary Dry Spinning of Regenerated Silk Fibroin Aqueous Solution. Mater. Sci. Eng. C.

[156] Wei W., Zhang Y., Shao H., Hu X. (2011). Posttreatment of the Dry-Spun Fibers Obtained from Regenerated Silk Fibroin Aqueous Solution in Ethanol Aqueous Solution. J. Mater. Res..

[157] Canetti M., Seves A., Secundo F., Vecchio G. (1989). CD and Small-Angle X-ray Scattering of Silk Fibroin in Solution. Biopolymers.

[158] Lee K.H., Baek D.H., Ki C.S., Park Y.H. (2007). Preparation and Characterization of Wet Spun Silk Fibroin/Poly(Vinyl Alcohol) Blend Filaments. Int. J. Biol. Macromol..

[159] Magoshi J. (1977). Physical Properties and Structure of Silk: 4. Spherulites Grown from Aqueous Solution of Silk Fibroin. Polymer.

[160] Li G., Yu T. (1989). Investigation of the Liquid-Crystal State in Silk Fibroin. Die Makromol. Chem. Rapid Commun..

[161] Kerkam K., Viney C., Kaplan D., Lombardi S. (1991). Liquid Crystallinity of Natural Silk Secretions. Nature.

[162] Knight D.P., Vollrath F. (1999). Liquid Crystals and Flow Elongation in a Spider’s Silk Production Line. Proc. R. Soc. London. Ser. B Biol. Sci..

[163] Willcox P.J., Gido S.P., Muller W., Kaplan D.L. (1996). Evidence of a Cholesteric Liquid Crystalline Phase in Natural Silk Spinning Processes. Macromolecules.

[164] Jenkins S., Thammongkol V., Polk M.B. (1998). Synthesis and Spinning of a Thermotropic Liquid Crystal Copolyester Containing a Semirigid Cycloaliphatic Spacer. J. Polym. Sci. Part. A Polym. Chem..

[165] Hyde S. (2002). CHAPTER 16 Identification of Lyotropic Liquid Crystalline Mesophases. Handbook of Applied Surface and Colloid Chemistry.

[166] Rey A.D., Herrera-Valencia E.E. (2012). Liquid Crystal Models of Biological Materials and Silk Spinning. Biopolymers.

